# 223. PIDS FEATURED ORAL ABSTRACT: Assessment of Bacterial Pathogens in Young Children with Acute Otitis Media: A Prospective Cohort Study in the United States (US)

**DOI:** 10.1093/ofid/ofae631.081

**Published:** 2025-01-29

**Authors:** Judith M Martin, Nader Shaikh, Kristin Yahner, Matthew C Lee, Jessica Weaver, Meghan White, Alejandro Hoberman

**Affiliations:** University of Pittsburgh, Pittsburgh, PA; University of Pittsburgh, Pittsburgh, PA; University of Pittsburgh, Pittsburgh, PA; University of Pittsburgh School of Medicine, Pittsburgh, Pennsylvania; Merck & Co., Inc., Rahway, New Jersey; Merck & Co., Inc., Rahway, New Jersey; University of Pittsburgh School of Medicine, Pittsburgh, Pennsylvania

## Abstract

**Background:**

Despite the success of pediatric pneumococcal conjugate vaccine (PCV) programs in the US, acute respiratory illness, including acute otitis media (AOM) due to Streptococcus pneumoniae (SPN) persists. PCV13 has been recommended for pediatric routine use since 2010. With recent adoption of higher-valency PCVs, there is a continued need to survey pathogens causing AOM in children.

**Table 1:**
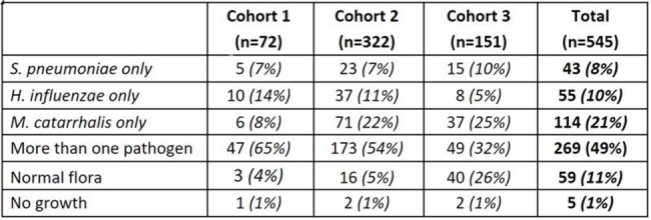
Bacterial Results of Nasal Specimens in Children 6-35 Months Diagnosed with Acute Otitis Media (Cohorts 1&2) or Upper Respiratory Infection (Cohort 3)

**Methods:**

Children aged 6 to 35 months diagnosed with AOM (Cohort 1 & 2) and upper respiratory infection (URI, Cohort 3) were enrolled from primary care and ENT practices in Pittsburgh, PA. Nasal specimens (nasopharyngeal or mid-turbinate) were collected for all children. Middle ear fluid (MEF) specimens were collected by tympanocentesis for children enrolled in Cohort 1. MEF and nasal specimens were cultured for SPN, Hemophilus influenzae (Hflu), and Moraxella catarrhalis (Mcat). Serotypes (ST) and susceptibility testing were determined for SPN isolates.

**Table 2:**
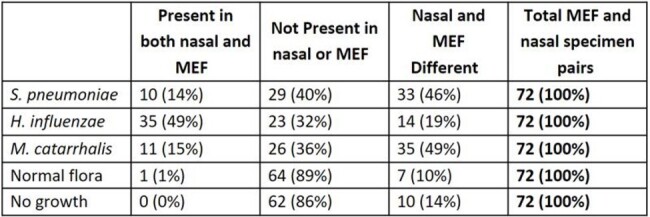
Correlation of Bacterial Cultures of Nasal and Middle Ear Fluid (MEF) Specimens (n=72 pairs) in Children 6-35 Months Diagnosed with Acute Otitis Media (Cohort 1)

**Results:**

From October 2019 to January 2024, 545 children were enrolled (Cohort 1: n=72; Cohort 2: n=322; Cohort 3: n=151). Among nasal specimens from children with AOM (n=394), SPN, Hflu and Mcat were detected in 47%, 44% and 70%, respectively. In MEF specimens from children with AOM (n=72), SPN, Hflu and Mcat were detected in 15%, 54% and 17% respectively. Nasal-MEF pairs were concordant for 79% (57/72). Children with Hflu had higher rates of nasal-MEF concordance than children with SPN or Mcat. In nasal specimens from children with URI (n=151), SPN, Hflu and Mcat were detected in 36%, 18% and 54%, respectively. In nasal specimens, co-detection of bacterial pathogens occurred more often in those with AOM (220/394, 56%) than URI (49/151, 32%). Of the SPN ST results available (nasal: n=174; MEF: n=8), 35B, 15B, 3, and 15C were most frequent; 8% of nasal and 50% of MEF were PCV13 STs. SPN was found simultaneously in the nasal-MEF pairs of 10/72 children, of which STs are available for 7 pairs; 6/7 pairs agreed, 1 disagreed (nasal:35B and MEF:3). SPN nonsusceptible to penicillin were uncommon (2% of nasal and 9% of MEF isolates).

**Figure 1:**
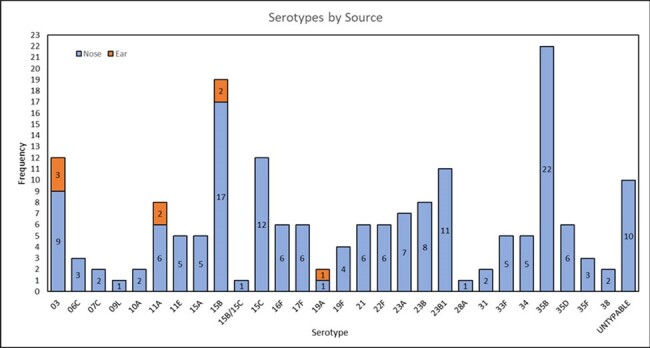
Distribution of S. pneumoniae Serotypes by Isolate Source

**Conclusion:**

STs 3 and 19A, included in PCV13, were responsible for half of SPN isolates found in MEF of children with AOM. Continued surveillance is still warranted to guide vaccine development and therapeutic decision-making for children.

**Disclosures:**

**Judith M. Martin, MD**, Centers for Disease Control and Prevention: funding to the institution to support this work|Centers for Disease Control and Prevention: funding to the institution for unrelated work|Merck, Sharp and Dhome: funding to the institution to support this work|Moderna: funding to the institution for unrelated work|NIH: funding to the institution for unrelated work **Matthew C. Lee, BA**, Centers for Disease Control and Prevention (CDC): Grant paid to the institution|Merck Sharpe & Dohme: Grant paid to the institution **Jessica Weaver, PhD, MPH**, Merck & Co. Inc.: Employment|Merck & Co. Inc.: Stocks/Bonds (Public Company) **Meghan White, PharmD**, Merck Sharp & Dohme, LLC: Employee|Merck Sharp & Dohme, LLC: Stocks/Bonds (Public Company) **Alejandro Hoberman, MD**, Kaizen Biosciences: Advisor/Consultant|Kaizen Biosciences: Pediatric oral suspension formulation of amoxicillin-clavulanate potassium and the method for use.|Kaizen Biosciences: Ownership Interest|Not licensed: Method and apparatus for aiding diagnosis of otitis media by classifying tympanic membrane images.

